# Retentions of the Saliva

**Published:** 1861-02

**Authors:** 


Retentions of the Saliva.—*Catheterism of the Duct of Steno upon a Patient Affected with Mumps.*—Translation by C.—The 28th November, 1859, I was called to M. W., aged twenty-two years, attacked twenty-four hours before with mumps on the right side. The parotid gland, hard and swollen, formed a relief behind the ascending branch of the inferior maxilla. It was painful to the pressure. Yet the patient could still open the mouth sufficiently to permit the orifice of the duct of steno to be distinguished. Around this orifice the mucous membrane was dry; the papilla at the top of which it opens, was projecting and distended. I introduced to a considerable distance into this duct a little silver probe, to which I had previously given a slight curve, so as more easily to pass round the edge of the masseter. The probe being drawn out there immediately issued a considerable quantity of viscous and ropy saliva. At the same time, the volume, as well as the consistence of the parotid very sensibly decreased.

In questioning the patient as to the manner in which this *parotiditis* could have been produced, I was not able to ascertain any other cause than a sudden transition from warm to cold. Now it is from the same cause that the formation of certain idiopathic tumors of the duct of steno is to be referred, which tumors give rise to an acumulation of saliva, then to an abscess, and at last to a salivary fistula. It was thus in the observation of Mours: "A young man of a delicate temperament, after a horseback ride during a cold night, was attacked with a fistular tumor in the middle of the left cheek." (*Mem. de l'Académie de Chirurgie, t.* ix, *p.* 66.) It was the same with two cases of salivary tumor, followed by fistula, which I have had opportunity to examine, and one of which I have published. (*Monit des Hôpitaux,* 15 November, 1853.) The impression of cold is then sufficient to produce, sometimes an engorgement of the parotid gland, and sometimes an accumulation of saliva in a portion of the duct of steno, with a distention of this part of the duct. Actual observation shows that, in similar cases, the *catheterism* of the duct of steno, performed near the beginning of the disease, will favor the cure by giving an outlet immediately to the accumulated saliva.—(*Répertoire medicale.*) *L'Art Dentain.*

